# Clinical features and potential risk factors for discerning the critical cases and predicting the outcome of patients with COVID‐19

**DOI:** 10.1002/jcla.23547

**Published:** 2020-08-29

**Authors:** Weili Wang, Zhongxiu Zhao, Xi Liu, Gang Liu, Dongjing Xie, Zhi Xu, Jinghong Zhao, Jingbo Zhang

**Affiliations:** ^1^ Department of Nephrology The Key Laboratory for the Prevention and Treatment of Chronic Kidney Disease of Chongqing Kidney Center of PLA Xinqiao Hospital Army Medical University (Third Military Medical University) Chongqing China; ^2^ Department of Ophthalmology Chongqing Aier‐Mega Eye Hospital Aier Eye Hospital Group Chongqing China; ^3^ Department of Gastroenterology Xinqiao Hospital Army Medical University (Third Military Medical University) Chongqing China; ^4^ Institute of Respiratory Diseases Xinqiao Hospital Army Medical University (Third Military Medical University) Chongqing China; ^5^ Department of Neurology Xinqiao Hospital Army Medical University (Third Military Medical University) Chongqing China

**Keywords:** clinical features, COVID‐19, critical patients, risk factors

## Abstract

**Objective:**

To investigate the clinical features and risk factors for discerning the critical and predicting the outcome of patients with COVID‐19.

**Methods:**

Patients who were admitted to the intensive care unit (ICU) department and general infection department of TaiKang Tongji (Wuhan) Hospital from February 10 to March 27, 2020, were included. Data on clinical features, complications, laboratory parameters, chest CT, nutrient requirement, and electrolyte imbalance were analyzed retrospectively.

**Results:**

A total of 123 (50 critical and 73 non‐critical) patients were enrolled. 65% of patients with comorbidities, hypertension (45.5%), diabetes (21.9%), 36.5% of patients had more than one comorbidity. The proportion of lymphocytes in critical patients was significantly lower than that of non‐critical patients. The proportion of patients with increased NLR, PLR, IL‐6, CRP levels, and chest CT score was significantly higher in the critical than that of non‐critical patients. The logistic regression analysis identified low lymphocyte count, high NLR, PLR, IL‐6, CRP levels, and CT score as independent factors for discerning critical cases and high NLR, PLR, IL‐6, and CT score could predict poor clinical outcome. Furthermore, we identified patients who needed nutrition support (HR 16.99) and with correction of electrolyte imbalance (HR 18.24) via intravenous injection were more likely to have a poor outcome.

**Conclusions:**

The potential risk factors of lower lymphocyte count, high levels of NLR, PLR, IL‐6, CRP, chest CT score, and the statue of nutrient requirement or electrolyte imbalance could assist clinicians in discerning critical cases and predict the poor outcome in patients with COVID‐19.

## Graphical abstract

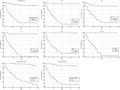

## 1 INTRODUCTION

In December 2019, an outbreak of an emerging disease associated with a novel coronavirus began in Wuhan, China. This novel virus shares about 79% and 50% of its genetic sequence with the coronaviruses responsible for severe acute respiratory syndrome (SARS) and the Middle East respiratory syndrome (MERS).^1^ It has been subsequently named the 2019 novel coronavirus disease (COVID‐19) by WHO.^2^ Currently, COVID‐19 is a global pandemic. Until May 21, 2020, there have been 4 864 881 confirmed cases of COVID‐19, including 321 818 deaths, reported to WHO. Most patients had mild symptoms or exhibited fever and dry cough, but some of them quickly developed into dyspnea, even acute respiratory distress syndrome (ARDS).

Accumulated evidence suggests that patients suffered from severe COVID‐19 with a dysregulation of the immune response.^3^ It is crucial to identify potential risk factors for discerning and prognosticating critical patients with COVID‐19. Recent researches reported that the neutrophil‐to‐lymphocyte ratio (NLR) or interleukin‐6 (IL‐6) might as independent risk factors of mortality for COVID‐19 patients.^2^, ^4^, ^5^ Whether other potential risk factors have prognostic value needs to be further elucidated. Here, we present clinic details of critical and non‐critical patients confirmed COVID‐19 and outcome. We aim to investigate the ability of IL‐6, C‐reactive protein (CRP), NLR, PLR, and the score of chest CT to serve as valuable risk factors of discerning and prognosticating critical patients. Moreover, considering the effects of nutritional status and the imbalance of internal environment on patients, we also evaluate the association of patients with electrolyte imbalance or needed nutrition support and outcome.

## 2 METHODS

### 2.1 Study design and participants

We performed this retrospective study on the clinical characteristics of laboratory‐confirmed patients with COVID‐19. Patients were admitted to the intensive care unit (ICU) department and general infection department of TaiKang Tongji (Wuhan) Hospital, a major institution for COVID‐19 treatment assigned by the People's Liberation Army of China. Cases with incomplete data will be excluded. A total of 123 cases that come from the community or nursing home from February 10 to March 27, 2020 were included. All cases were positive of SARS‐CoV‐2 nucleic acid RT‐PCR and diagnosed according to guidelines of COVID‐19 in China. Critical patients and non‐critical patients (including severe and mild patients) were categorized based on the 7th edition of the Chinese National Health Commission,^6^ meeting any of the following criteria: (a) respiratory failure required mechanical ventilation; (b) the patient was prone to shock; (c) the patient with multiple organ failure required ICU treatment. This retrospective observational study was approved by the institutional Research Ethics Committee of the Chinese People's Liberation Army Joint Logistic Support Force. The outcome of cases was defined as patients who received mechanical ventilation or all‐cause death.

### 2.2 Data collection

Information about demographic characteristics, symptoms, laboratory parameters, chest computed tomographic (CT) images, and treatments was all collected and reviewed from the electronic medical record system (EMRS). Peripheral venous blood samples, which were analyzed within two h, were assessed at the central laboratory of TaiKang Tongji (Wuhan) Hospital following the standard operative procedures. The CT scans were performed in the same institution. The score of chest CT was evaluated according to the following criteria: (a) no obvious abnormality; (b) unilateral ground glass infiltration or consolidation <50% area; (c) unilateral ground glass infiltration or consolidation ≥50% area; (d) bilateral ground glass infiltration or consolidation <50% area; (e) bilateral ground glass infiltration or consolidation ≥50% area. Patients‐needed nutritional support was defined as intravenous glucose, amino acid, or albumin supplementation ≥3 days. Electrolyte imbalance was defined as requiring intravenous infusion for correcting electrolyte imbalance ≥3 days. The data used in the study were anonymous. Data of patients were checked by two independent investigators.

### 2.3 Statistical analysis

Continuous variables were appropriately expressed as median (IQR) or mean (SD). Categorical variables were presented as median (IQR) or n (%). Continuous and categorical variables were compared by the Mann‐Whitney *U* test, χ^2^ test, or Fisher's exact test appropriately. The predictive value of parameters was evaluated by measuring the receiver operating characteristic curve (ROC). The optimal critical value of each parameter was determined by calculating the Youden index. Logistic regression analysis (the Enter method) was performed to determine possible risk factors associated with discerning critical patients or an outcome. *P* < .05 was considered statistically significant. All these statistical analyses were performed using the SPSS 23.0 or MedCalc 19.

## 3 RESULTS

### 3.1 Demographic data, clinical symptoms, treatments, and outcome of patients with COVID‐19

A total of 123 patients with COVID‐19 were included in this study. The detailed information of study subjects is shown in Table 1. In the study population, 50 patients (40.7%) were eligible for critical illness; 73 patients (59.3%) were eligible for non‐critical illness. The median age was 68 years old, and the maximum age was 96 years old. The median age was significantly higher in the critical group than those of the non‐critical group (*P* < .001). Patients over 60 or 75 years old between groups were also different (*P* = .000, *P* < .000, respectively). There was no gender difference between the two groups (*P* = .203). Overall, the most common initial symptoms were fever (61.7%) and cough (53.6%). 65.1% of patients had comorbidities, 36.5% of patients had more than one, including hypertension (45.5%), diabetes (21.9%), heart disease (17.1%), respiratory diseases (9.7%), chronic kidney disease (8.1%), and post‐stroke (8.1%). Of all cases, five had tumors. Patients with comorbidities in the critical group were more than in the non‐critical group (*P* < .000).

**Table 1:** Demographic, clinical, treatment, and outcome of patients hospitalized with COVID‐19

Variable	Total (n = 123)	Critical (n = 50)	Non‐critical (n = 73)	Statistic test
Demographic				
Age, y, median (IQR)	68 (56.5‐78.0)	79.5 (68‐87)	61 (50‐68)	*P* < .001
Age ≥60 y	83 (67.4%)	44 (88%)	39 (53.4%)	*P* = .000
Age ≥75 y	38 (30.8%)	31 (60%)	7 (9.5%)	*P* < .000
Sex (male/female, %)	60 (48.7%)/63 (51.3%)	28 (56.0%)/22 (44.0%)	32 (43.8%)/41 (56.2%)	*P* = .203
Symptoms				
Fever	76 (61.7%)	33 (66.0%)	43 (58.9%)	*P* = .428
Cough	66 (53.6%)	22 (44.0%)	44 (60.2%)	*P* = .077
Dyspnea	44 (35.7)	24 (48%)	20 (27.3%)	*P* = .019
Fatigue	14 (11.3)	4 (8%)	10 (13.6%)	*P* = .330
Vomiting	2 (1.6%)	0	2 (2.7%)	*P* = .239
Diarrhea	10 (8.1%)	2 (4%)	8 (10.9%)	*P* = .167
Comorbidities	80 (65.0%)	46 (92%)	34 (46.6%)	*P* < .000
Hypertension	56 (45.5%)	31 (62%)	25 (34.2%)	*P* = .002
Diabetes	27 (21.9%)	11 (22%)	16 (21.9%)	*P* = .991
Heart disease	21 (17.1%)	14 (28%)	7 (9.5%)	*P* = .007
COPD	12 (9.7%)	8 (16%)	4 (5.4%)	*P* = .054
Asthma	1 (0.8%)	0	1 (1.4)	*P* = .407
Chronic kidney disease	10 (8.1%)	6 (12%)	4 (5.4%)	*P* = .195
Anemia	3 (2.4%)	3 (6%)	0	*P* = .034
Tumor	5 (4%)	2 (4%)	3 (4.1%)	*P* = .976
Using immunosuppressants	1 (0.8%)	1 (2%)	0	*P* = .227
Post‐stroke	10 (8.1%)	10 (8.1%)	0	*P* = .000
Dementia	3 (2.4%)	3 (6%)	0	*P* = .034
More than one comorbidity	45 (36.5%)	29 (58%)	16 (21.9%)	*P* < .000
Outcome				
Days from onset to admission median (IQR)	20 (10‐30)	14 (6‐30)	20 (11‐30)	*P* = .296
Received mechanical ventilation	22 (17.8%)	22 (44%)	0	*P* < .000
Days (from admission), median (IQR)	4 (2‐7)	4 (2‐7)	—	
All‐cause death	17 (13.8%)	16 (32%)	1 (1.4%)	*P* < .000
Days (from admission), mean (SD)	11.76 (6.29)	11.80 (6.49)	—	
Treatments				
Antibiotic	47 (38.2%)	40 (80%)	7 (9.5%)	*P* = .578
Correction of electrolyte Imbalance	44 (35.7%)	40 (80%)	4 (5.4%)	*P* < .000
Needed nutrition support	48 (39%)	43 (86%)	5 (6.8%)	*P* < .000
CRRT	3 (2.4%)	3 (6%)	0	*P* < .039
ECMO	1 (0.8%)	1 (2%)	0	*P* = .227

Abbreviations: COPD, chronic obstructive pulmonary disease; CRRT, continuous renal replacement therapy; ECMO, extracorporeal membrane oxygenation.

### 3.2 Laboratory parameters and chest CT score between critical and non‐critical patients

The level of laboratory parameters and the score of CT, white blood cells, neutrophils, NLR, PLR, CRP, IL‐6, PCT, serum sodium concentration, PT, D‐dimer, and CT score, were significantly higher in the critical group than those of non‐critical group. On the contrary, the level of lymphocytes, uric acid, and albumin in the critical group was lower in the non‐critical group (Table 2).

**Table 2:** Laboratory results and radiological feature of COVID‐19 patients

Parameters	Total	Critical	Non‐critical	Statistic test
Laboratory results				
White blood cells × 10^9^/L, median (IQR) (reference range, 3.5‐9.5)	6.57 (4.86‐8.13)	8.13 (5.42‐11.85)	5.88 (4.82‐7.03)	*P* = .000
Neutrophils × 10^9^/L, median (IQR) reference range, 1.8‐6.3)	3.97 (2.87‐5.73)	6.41 (4.18‐9.82)	3.29 (2.46‐4.21)	*P* = .000
Lymphocytes × 10^9^/L, median (IQR) (reference range, 1.1‐3.2)	1.48 (0.79‐1.97)	0.67 (0.50‐0.95)	1.84 (1.52‐2.16)	*P* = .000
Platelet × 10^9^/L, mean (SD) (reference range, 125‐350)	221.78 (85.34)	206.82 (88.69)	232.03 (82.01)	*P* = .066
Neutrophil‐to‐lymphocyte ratio, median (IQR)	2.18 (1.58‐7.49)	9.30 (5.82‐15.10)	1.83 (1.40‐2.13)	*P* = .000
Platelet‐to‐lymphocyte ratio median (IQR)	148.6 (112.14‐243.17)	248.60 (176.29‐404.44)	120.13 (97.37‐149.57)	*P* = .000
C‐reactive protein, mg/L, median (IQR) (reference range, 0‐10)	3.27 (0.50‐25.48)	28.95 (13.39‐66.85)	0.50 (0.50‐2.10)	*P* = .000
Interleukin‐6, pg/mL, median (IQR) (reference range, 0‐7)	6.34 (2.35‐36.90)	44.94 (14.94‐94.98)	2.78 (1.50‐4.28)	*P* = .000
Procalcitonin, ng/mL, median (IQR) (reference range, 0‐0.05)	0.057 (0.036‐0.121)	0.122 (0.087‐0.247)	0.037 (0.029‐0.053)	*P* = .000
Na+, mmol/L, median (IQR) (reference range, 137‐147)	139.60 (137.45‐141.20)	138.20 (134.20‐141.10)	140.10 (138.40‐141.20)	*P* = .008
Creatinine, μmol/L, median (IQR) (reference range, 64‐104)	56.00 (44.32‐73.39)	54.96 (40.40‐74.64)	57.05 (45.21‐72.62)	*P* = .563
Urea nitrogen, mmol/L, median (IQR) (reference range, 2.8‐7.2)	5.43 (4.23‐6.69)	5.78 (4.31‐7.75)	5.19 (4.04‐6.23)	*P* = .070
Uric acid, μmol/L, median (IQR) (reference range, 142‐420)	262.28 (201.30‐342.94)	209.85 (147.55‐310.67)	289.30 (240.40‐346.42)	*P* = .001
Albumin, g/L, median (IQR) (reference range, 35‐55)	37.00 (31.27‐40.64)	31.17 (28.75‐33.82)	39.55 (37.12‐41.77)	*P* = .000
PT, s, median (IQR) (reference range, 9.4‐12.5)	12.20 (11.30‐13.40)	13.50 (12.30‐14.60)	11.50 (11.05‐12.35)	*P* = .000
APTT, s, median (IQR) (reference range, 25.1‐36.5)	31.50 (29.10‐33.70)	31.35 (27.00‐34.80)	31.65 (29.70‐33.35)	*P* = .482
D‐dimer, ng/mL, median (IQR) (reference range, 0‐243)	380.50 (176.50‐944.00)	685.00 (423.00‐2149.00)	169.00 (108.00‐209.00)	*P* = .000
Radiological features				
Score of chest CT	3 (2‐4)	4 (4‐5)	2 (1‐3)	*P* = .000

Abbreviations: APTT, activated partial thromboplastin time; PT, prothrombin time.

ROC curve analysis was used to discern critical and non‐critical patients with COVID‐19. The areas under the curve (AUC) of lymphocyte, NLR, PLR, IL‐6, CRP, and CT score were 0.914, 0.965, 0.850, 0936, 0.934, and 0.930, respectively. The optimal cutoff values relative to the preceding indicators were 1.01, 4.16, 172.44, 6.49, 4.27, and 3 for lymphocyte, NLR, PLR, IL‐6, CRP, and CT score, respectively (Table 3). Considering that the parameters of NLR and PLR were calculated based on the lymphocyte ratio, we further compared the differences of ROC curves among the three parameters. AUC of lymphocyte was larger than AUC of PLR (*P* = .027, *Z* = 2.214, 95% CI 0.007‐0.121), and AUC of NLR was larger than AUC of PLR (*P* = .001, *Z* = 3.401, 95% CI 0.049‐0.182). There was no difference between lymphocyte and NLR (*P* = .082).

**Table 3:** ROC analysis of each parameter to discern critical patients with COVID‐19

Parameters	AUC	Optimal cutoff value	Sensitivity %	Specificity %	95% CI	*P* value
Lymphocyte	0.914	1.01	80	97.26	0.850‐0.957	.000
NLR	0.965	4.16	84	97.26	0.916‐0.990	.000
PLR	0.850	172.44	78	80.82	0.774‐0.908	.000
IL‐6	0.936	6.49	94	84.06	0.876‐0.973	.000
CRP	0.934	4.27	88	88.73	0.874‐0.971	.000
CT score	0.930	3	79.49	93.15	0.866‐0.969	.000

We further used ROC curve analysis to predict the outcome of COVID‐19 patients. The AUC of lymphocyte, NLR, PLR, IL‐6, CRP, and CT score was 0.825, 0.913, 0.788, 0.895, 0.866, and 0.823, respectively. The optimal cutoff values relative to the preceding indicators were 1.01, 4.31, 189.11, 10.63, 3.3, and 3 for lymphocyte, NLR, PLR, IL‐6, CRP, and CT score, respectively (Table 4). On the pairwise comparison of ROC curves, the AUC of NLR is larger than AUC of lymphocyte (*P* = .033, *Z* = 2.133, 95% CI 0.007‐0.169). The AUC of NLR is larger than AUC of PLR (*P* = .004, *Z* = 2.847, 95% CI 0.039‐0.210). There is no significant difference between the AUC of lymphocyte and PLR (*P* = .392).

**Table 4:** ROC analysis of each parameter to predict outcome with COVID‐19 patients

Parameters	AUC	Optimal cutoff value	Sensitivity %	Specificity %	95% CI	*P* value
Lymphocyte	0.825	1.01	80.65	81.52	0.746‐0.887	.000
NLR	0.913	4.31	90.32	83.70	0.848‐0.956	.000
PLR	0.788	189.11	77.42	75.00	0.705‐0.857	.000
IL‐6	0.895	10.63	93.55	76.14	0.826‐0.944	.000
CRP	0.866	3.3	100	68.13	0.792‐0.921	.000
CT score	0.823	3	70	76.09	0.740‐0.889	.000

### 3.3 Logistic regression analysis of the association of lymphocyte counts, NLR, PLR, IL‐6, CRP, CT score, need nutrition support, electrolyte imbalance, and critical patients during hospitalization or outcome

To further identify the risk factors that may discern critical patients during COVID‐19 progression and evaluate the outcome, the logistic regression analysis was conducted. Given that the significant differences of age and comorbidities, but no gender, the crude odds ratio (OR), and the adjusted OR with age and comorbidities were calculated. Results showed that seven parameters, the NLR, PLR, IL‐6, CRP, CT score, patients who need nutrition support, and electrolyte imbalance, were positively correlated with the risk of critical patients. Still, the lymphocyte count was a negative correlation (Table 5). On the logistic regression analysis of the association of parameters and outcome, including patients who received mechanical ventilation or all‐cause death of cases, NLR, PLR, IL‐6, CT score, patients who need nutrition support and with electrolyte imbalance were positively correlated with the risk of the outcome. Conversely, lymphocyte count was negatively associated with the outcome (Table 6).

**Table 5:** Logistic regression analysis of the association of parameters and critical patients during hospitalization

Parameters	OR (95% CI)	*P* value	Adjusted OR (95% CI)	*P* value
Lymphocyte	0.169 (0.083‐0.342)	.000	0.331 (0.161‐0.681)	.002
NLR	2.493 (1.743‐3.567)	.000	2.142 (1.469‐3.124)	.000
PLR	1.016 (1.009‐1.022)	.000	1.014 (1.007‐1.021)	.000
IL‐6	1.064 (1.033‐1.095)	.000	1.043 (1.015‐1.072)	.002
CRP	1.134 (1.073‐1.199)	.000	1.123 (1.057‐1.193)	.000
CT score	16.707 (5.676‐49.177)	.000	22.038 (4.783‐101.538)	.000
Need nutrition support	83.542 (24.924‐280.027)	.000	38.690 (9.759‐153.383)	.000
Electrolyte imbalance	69.000 (20.305‐234.470)	.000	28.984 (7.760‐108.256)	.000

Note

Adjusted OR means adjustment for age and comorbidities.

**Table 6:** Logistic regression analysis of the association of parameters and the outcome

Parameters	OR (95% CI)	*P* value	Adjusted OR (95% CI)	*P* value
Lymphocyte	0.409 (0.217‐0.770)	.005	0.826 (0.477‐1.430)	.495
NLR	1.219 (1.110‐1.3387)	.000	1.156 (1.070‐1.250)	.000
PLR	1.006 (1.003‐1.009)	.000	1.005 (1.002‐1.008)	.001
IL‐6	1.023 (1.012‐1.034)	.000	1.014 (1.004‐1.025)	.005
CRP	1.024 (1.011‐1.037)	.000	1.013 (0.999‐1.027)	.063
CT score	3.474 (1.930‐6.254)	.000	2.806 (1.466‐5.371)	.001
Need nutrition support	33.60 (9.252‐22.017)	.000	15.697 (3.594‐68.558)	.000
Electrolyte imbalance	21.377 (7.209‐63.388)	.000	8.783 (2.557‐30.166)	.000

Note

Adjusted OR means adjustment for age and comorbidities.

### 3.4 Kaplan‐Meier curves of outcome between critical and non‐critical cases

**Figure 1:**
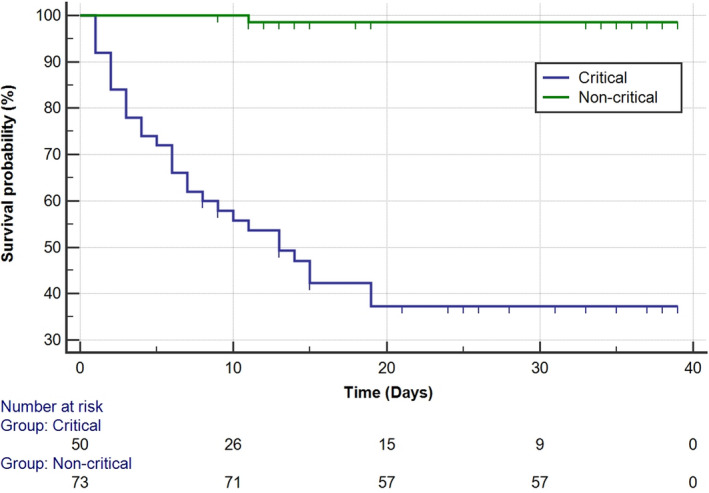
Mean survivals of critical and non‐critical patients with COVID‐19

The median follow‐up time was 33 days, a minimum of 1 day, and a maximum of 39 days. Kaplan‐Meier analysis showed that the mean survivals of critical and non‐critical patients were 19.21 ± 2.30 and 38.60 ± 0.39, with a significant statistical difference (HR 20.69, 95% CI 9.64‐44.41, *P* < .000) (Figure 1). To verify risk factors for distinguish outcome of COVID‐19 patients, cases were classified into high and low groups according to the optimal threshold of these parameters. Results showed that patients with higher value of NLR (*P* < .000, HR 20.72, 95% CI 9.460‐45.381), PLR (*P* < .000, HR 7.09, 95% CI 3.352‐15.001), IL‐6(*P* < .000, HR 13.73, 95% CI 6.508‐28.981), CRP (*P* < .000), CT score (*P* < .000), or who needed nutrition support (*P* < .000, HR 16.99, 95% CI 7.862‐36.746) or with electrolyte imbalance (*P* < .000, HR 18.24, 95% CI 8.195‐40.592) than the optimal threshold had a worse outcome, with all‐cause death or received mechanical ventilation. Whereas, lymphocyte count (*P* < .000, HR 0.065, 95% CI 0.029‐0.147) acted as a protective factor, showed that patients with higher lymphocytes had a better outcome.

## 4 DISCUSSION

COVID‐19 has rapidly spread throughout the world. COVID‐19 causes a wide spectrum of clinical manifestations, from asymptomatic or mild‐symptom to fatal viral pneumonia and multiple organ failure. Previous researches showed that 13%‐26% of patients required ICU care, and mortality was 4.3%‐15%.^7^, ^8^ 123 COVID‐19 patients in our retrospective study, 50 critical cases were all from the ICU department, and the total in‐hospital mortality was 13.8%. Our data showed that the potential risk factors, including low lymphocyte count, high levels of NLR, PLR, IL‐6, CRP, chest CT score, the statue of nutrient requirement, and electrolyte imbalance, could assist clinicians in discerning critical cases and predict the poor outcome in patients with COVID‐19. The critical cases should be discerned as early as possible, risk stratification management will be applied quickly, and more critical patients might be saved. Furthermore, the imbalance of nutrient requirements and internal environment could also be associated with the critical and outcome of patients, especially to critical cases, 80% with electrolyte imbalance, and 86% needed intravenous nutrition support.

Dysregulation of immune response plays an important role in patients with COVID‐19. Results of epidemiological studies are suggestive of lymphopenia and hypercytokinemia, which may act as hallmarks of COVID‐19.^3^, ^9^ Lymphopenia has been observed in most patients. In this study, critical patients with a lower level of lymphocytes than non‐critical patients (0.67 (0.50‐0.95) vs 1.84 (1.52‐2.16), *P* = .000) had a higher risk for outcome after adjustment for other cofounders. Some explanations are posited to explain the lymphopenia caused by SARS‐CoV‐2. First, the coronavirus can directly infect T cells but fail to replicate within the cells. Second, inhibitory cytokines are released by macrophages or lung epithelial cells that cause T‐cell apoptosis or prevent their proliferation.^10^, ^11^ Same as acquired immunity, the innate immune response to SARS‐CoV‐2 infection is also overly exuberant.^12^ Neutrophils are the first line of innate immune defense. Under an inflammatory environment, neutrophils are activated by proinflammatory cytokines or chemokines and also able to secrete them to amplify inflammation process interaction with other immune cells.^13^ Platelets play a crucial role in the inflammatory response to recruit neutrophils and other inflammatory cells to the site of injury.^14^ The NLR and PLR, reflecting different kinds of immune states in the body, been proposed as novel biomarkers for inflammation diseases.^2^, ^15^ In rheumatoid arthritis (RA), NLR and PLR were higher than healthy controls and correlated with disease activity in patients. Although CRP is frequently evaluated in clinical practice to assess the activity of rheumatic diseases, NLR was shown to be more valuable than CRP to assess disease activity in RA.^16^ The increase of neutrophils and platelets may be due to anti‐apoptotic cytokines and stimulation by some pro‐inflammatory cytokines.^17^ In our study, white blood cells, procalcitonin, NLR, and PLR in critical were significantly higher than in non‐critical patients. In contrast, most critically patients (80%) are treated with antibiotics in our study. This phenomenon reflected the possibility of other bacterial infections in critical patients based on T‐cell exhaustion. On the pairwise comparison of AUC, NLR is superior to lymphocyte for predicting the outcome of COVID‐19 patients, but no differences between two factors for the discerning critical population.

COVID‐19 associates states of both immunodeficiency and hyperinflammation. The overly exuberant inflammation situations will eventually lead to critical conditions, which termed “cytokine storm”.^3^ Serum CRP usually increases rapidly and significantly during acute inflammatory responses in COVID‐19. IL‐6, a proinflammatory mediator, may further fuel the vicious cycle of releasing cytokines.^18^ Previous studies confirmed that both CRP and IL‐6 were elevated in acute phase reactants in patients with COVID‐19.^5^, ^19^ Here, we found that these two factors were higher and closely linked to critical disease. Patients with CRP > 4.27 mg/L or IL‐6 > 6.49 pg/mL were more likely to develop critical disease. IL‐6 > 10.63 pg/mL might indicate a poor outcome for patient.

In addition, we attempted to explore the relationship between CT score and critically patients. By scoring based on infiltration and consolidation imaging, we found that the lung injury of critical patients was more severe than that of non‐critically patients. Patients with scores three or above had a worse outcome.

Furthermore, considering the effects of nutritional status and homeostasis on the disease, we evaluated the patient who needed nutrition support and corrected electrolyte imbalance via intravenous injection ≥3 days. Another study confirmed that screening for patients with COVID‐19 who are at nutritional risk, as well as in need of additional nutritional intervention, is associated with their outcomes.^20^ Our results showed that critical patients are more likely to rely on intravenous nutrition support and corrected electrolyte imbalance than non‐critical patients, which means for some frail or elder patients, home isolation without hospital treatment may have a high risk.

This study has some limitations, including the number of cases was small and clinical data were limited. Some relevant data such as PT, APTT, D‐dimer were not complete and unable to be included in the risk factor analysis.

## 5 CONCLUSION

In conclusion, the lower lymphocyte count and the higher levels of NLR, PLR, CRP, IL‐6, and CT score have a significant correlation with the severity of COVID‐19, which can be used as useful risk factors to discern critical patients or predict disease outcome. Moreover, nutritional risk and electrolyte imbalance can also be considered as evaluation indicators for patients with COVID‐19.

## CONFLICT OF INTEREST

The authors declared that there is no conflict of interest.

## AUTHOR CONTRIBUTIONS

Weili Wang, Zhi Xu, Jinghong Zhao, and Jingbo Zhang designed the study. Weili Wang, Xi Liu, Gang Liu, and Dongjing Xie collected and organized data the data. Weili Wang and Zhongxiu Zhao analyzed and interpreted the data. Weili Wang and Jingbo Zhang wrote this manuscript and all the authors revised it.

## Ethical Approval

This retrospective observational study was approved by the institutional Research Ethics Committee of the Chinese People's Liberation Army Joint Logistic Support Force.
